# The Effect of Copper Salts on Bioactive Compounds and Ultrastructure of Wheat Plants

**DOI:** 10.3390/molecules27154835

**Published:** 2022-07-28

**Authors:** Otilia Culicov, Adina Stegarescu, Maria-Loredana Soran, Ildiko Lung, Ocsana Opriș, Alexandra Ciorîță, Pavel Nekhoroshkov

**Affiliations:** 1 Joint Institute for Nuclear Research, 6 Joliot-Curie, 1419890 Dubna, Russia; culicov@nf.jinr.ru (O.C.); p.nekhoroshkov@gmail.com (P.N.); 2 National Institute for Research and Development in Electrical Engineering ICPE-CA, 313 Splaiul Unirii, 030138 Bucharest, Romania; 3 National Institute for Research and Development of Isotopic and Molecular Technologies, 67-103 Donat, 400293 Cluj-Napoca, Romania; loredana.soran@itim-cj.ro (M.-L.S.); ildiko.lung@itim-cj.ro (I.L.); ocsana.opris@itim-cj.ro (O.O.); alexandra.ciorita@itim-cj.ro (A.C.); 4 Faculty of Biology and Geology, Babeș-Bolyai University, 5-7 Clinicilor, 400006 Cluj-Napoca, Romania

**Keywords:** copper, wheat, assimilating pigments, polyphenols, antioxidant activity, ultrastructure, elemental content, NAA, TEM, EDX

## Abstract

Abiotic stress agents, among them metal stress, can cause oxidative damage to plant cells. In defense, plants can increase the production of secondary metabolites in order to mitigate the harmful effects caused by them. The purpose of this work was to evaluate the effect of two types of copper salts (CuSO_4_ and Cu(NO_3_)_2_), added in two different amounts in soil (150 mg/kg, respectively 300 mg/kg), on assimilating pigments, total polyphenols, antioxidant activity and the elemental composition of wheat. The obtained results were compared with those from control plants grown in the same conditions but without copper salts. The amount of assimilating pigments, total polyphenols, and antioxidant activity respectively increases or decreases in the plants treated with copper salts compared to the control depending on the stage of development of the plant. No significant damage induced in the leaves of the wheat plants treated with the selected salts was observed following the TEM analysis. In six-week-old plants it was observed by EDX analysis that the salts are transformed into nanoparticles. The bioactive compounds, elemental composition and their interaction is influenced by concentration of metal’s salt, type of salt and exposure period.

## 1. Introduction

Copper is an essential micronutrient that is required for healthy growth and takes part in redox reactions, electron transfers and other critical metabolic processes in plants [1,2,3]. Nitrogen (N), also a critical element for plant growth and development [4,5], is absorbed as (NO_3_)^−^, which affects plant growth and contributes to secondary metabolite accumulation [6]. Nitrogen as (NO_3_)^−^ affects not only plant growth but also the nutritional quality of higher plants [6,7], influencing both the primary and secondary metabolic pathways and thus secondary plant metabolites accumulation [8]. The nitrogen deficient plants increase the availability of ammonia leading to the accumulation of the amount of phenolic compounds [9,10].

Copper (II) sulfate (cupric sulfate) is used in agriculture as a pesticide and fungicide to protect plants from bacterial disease and to control microbial activity, while cupric nitrate is used either as a fungicide or as an herbicide [10].

Copper plays an essential role in the synthesis of phenolic compounds, and its deficiency can decrease phenolics in the plants [10]. However, copper at high concentrations disturbs photosynthetic and mitochondrial electron transport, nitrogen assimilation, cell wall metabolism, affects different parameters of plant metabolism as dry mass accumulation, chlorophyll, water content and the balance in macro- and micronutrient levels. The toxicity of copper depends not only on the metal concentration and exposure duration but the development stage and physiological state of plants [11] as well.

Mwamba and his collaborators studied the physio-chemical changes in *Brassica napus* plants threat with different metal concentrations (0, 50, 200 µM), including copper, during fifteen days of exposure, focusing on the interactive effects of these metals on metal accumulation. They report a synergistic effect at low metal concentrations, where Cd effectively induced Cu bioaccumulation. In the plants treated only with copper, a lower accumulation was observed in the tissues than in the case of the plants treated only with Cd, but nevertheless Cu caused reduces the plant growth and photosynthetic capacity and also induced oxidative stress and damaged of the ultrastructure [12,13,14].

Sonmez et al. [15] investigated the effects of CuSO_4_·5H_2_O on the yield and growth of tomato plants. Two cupric fungicides (Cu oxychloride and Cu salts of fatty and rosin acids) were applicated to a calcareous soil and to leaves in three different levels (0, 1000 and 2000 mg Cu kg^−1^). The high concentration of Cu applied to the soil and leaves led to a weaker development of the tomato plants. Also, foliar application of copper sulfate (0.5 g L^−1^) increased dry matter peppermint yield up to 58% higher than in control and essential oil content and composition, respectively [16].

E. G. Krylova also investigates the effect of heavy metals in various concentrations on the growth and development of seedlings of *Bidens radiata* Thuill. The salts taken into account were nickel sulfate and copper sulfate and study the toxic effect of various concentrations of them on *Bidens radiata* Thuill and compare their resistance to the previously studied *B. tripartita*, *B. cernua*, and *B. frondosa*. She concludes that nickel sulfate is more toxic than copper sulfate for the growth and development of seedlings of *B. radiata* and produces significant damage at 10–50 mg/L in the morphometric parameters of seedlings of this species. Both metals and salts have a toxic effect manifested in the reduction of the main root. The *B. radiata* species is more resistant to the effects of copper than other species of this class [17,18,19].

Since plants are sensitive organisms, they possess a number of defence strategies. One of these strategies consists of the synthetization and accumulation of a spectrum of secondary metabolites such as phenolics, saponins, cyanogenic glycosides, cyclic hydroxamic acids, etc. They contribute to the specific odors, tastes and colors to plants and have protective functions in response to stress conditions. In the plants subjected to environmental stresses, including heavy metals, the accumulation of secondary metabolites often occurs. Therefore, the understanding of the effect of different copper source additions on the growth of crop plants has great importance. Jańczak-Pieniąźek et al., in 2022 released a study about the impact of the application of a quercetin—copper complex on *Triticum aestivum* L. growing under saline conditions. Stress on plants due to their growth in saline soil can lead to unfavorable changes in the photosynthesis process and to a decrease in plant productivity. For this reason, it is necessary to use biologically active substances, like quercetin, that reduce the effects of this stress. The foliar application of quercetin—copper solutions improved the values of photosynthesis process in the control plants in direct proportion to the concentrations of the complex applied. After the plants were sprayed to reproduce the salt stress can be observed an improvement in the values of physiological parameters. By salt stress application, an increase of level for ROS parameters and a perturbation of the activity of the antioxidant enzymes were observed. These decreased after applications of quercetin-copper solutions. The presented method can be a good solution for the implementation of sustainable agricultural practices [20].

Thus, the present study will investigate the bioactive compounds and the elemental content from the wheat plants treated with different copper sources and different concentrations.

The main objectives of this paper were to evaluate the influence of the copper (II) sulfate and copper (II) nitrate on the bioactive compounds from plants, on the ultrastructure of leaves and, on the other side, to study the impact on element distribution and transport capacity of plants.

## 2. Results

### 2.1. Analysis of Studied Bioactive Compounds

#### 2.1.1. Determination of Assimilating Pigments

**Figure 1:**
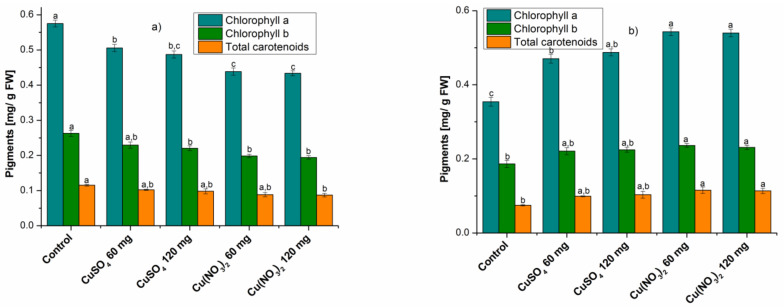
Comparative diagram of the pigment content; (**a**) at three weeks after sowing and (**b**) at six weeks after sowing. Each data point is the mean ± the standard error of the mean of three independent replicates experiments; different letters mean significant differences between the treatment and the control plants as determined by Tukey’s test (*p* < 0.05).

The amount of assimilating pigments in the studied plants is presented in Figure 1.

In the case of plants harvested three weeks after sowing, the amount of chlorophylls and total carotenoids was higher in the control plants compared to those treated. In addition, in the plants treated with CuSO_4_, the amount of pigments was higher than in the case of plants treated with Cu(NO_3_)_2_. Taking into account the amount of added salt to the soil, no significant differences were found in either of the two salts.

In plants harvested six weeks after sowing, the amount of chlorophyll a, chlorophyll b and total carotenoids was lower in the control plants than in the treated ones. Comparing the plants treated with the two copper salts, it was observed that the amount of pigments was lower in the case of plants treated with CuSO_4_ than in those treated with Cu(NO_3_)_2_, with no significant differences in the amount of salt added in soil.

A previous study concluded that at copper concentrations greater than 300 μm, the amount of chlorophyll decreases in tea plants [21]. Furthermore, a high concentration of CuSO_4_ exerted a considerable negative effect on chlorophylls and carotenoids in *Citrus aurantium* L. [22], on chlorophyll in *Camelia sinensis* [23], and some *Capsicum frustescens* varieties [24].

#### 2.1.2. Determination of Total Phenolic Content

**Figure 2:**
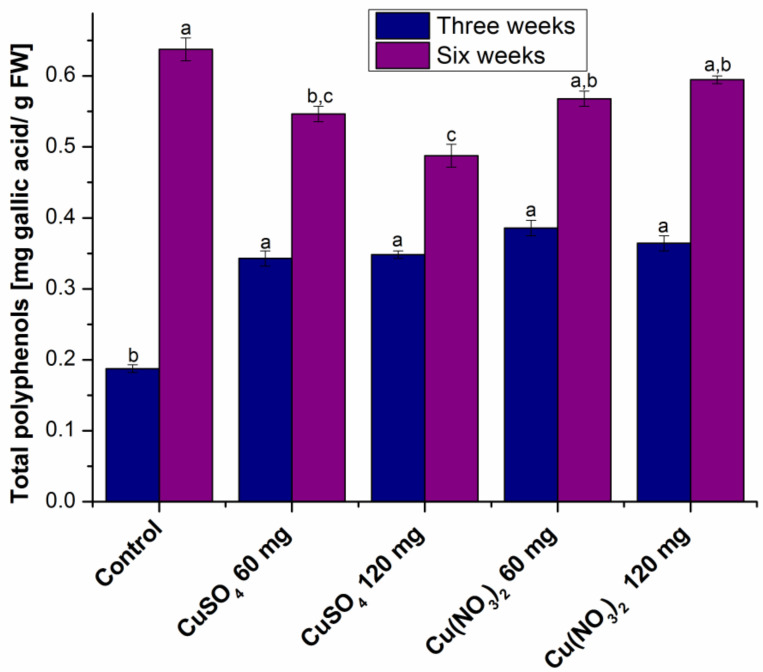
Comparative diagram of the total amount of polyphenolic compounds. Each data point is the mean ± the standard error of the mean of three independent replicate experiments; different letters mean significant differences between the treatment and the control plants, as determined by Tucky’s test (*p* < 0.05).

The amount of polyphenolic compounds expressed as mg gallic acid/g fresh weight (FW) is presented in Figure 2.

It can be seen that in the first series of plants, which were harvested three weeks after sowing, the amount of polyphenols in the treated plants is higher than in the control. Comparing the plants treated with the two copper salts, it was found that there are no major differences in terms of copper salt and the salt amount added to the soil.

In series II, in plants harvested six weeks after sowing, the amount of polyphenols in the treated plants was lower than in the control ones. Regarding the type of salt, plants treated with Cu(NO_3_)_2_ have higher amounts of polyphenols compared to those treated with CuSO_4_. In plants treated with the same salt, with the increasing of the CuSO_4_ amount, the quantity of polyphenols decreases, while in the case of Cu(NO_3_)_2_ it increases.

Heavy metals affect the plants and they develop a series of tolerance mechanisms to adapt [25]. Thus, in response to abiotic stress there was an increase in the amount of polyphenols [26].

#### 2.1.3. Determination of Total Antioxidant Capacity

**Figure 3:**
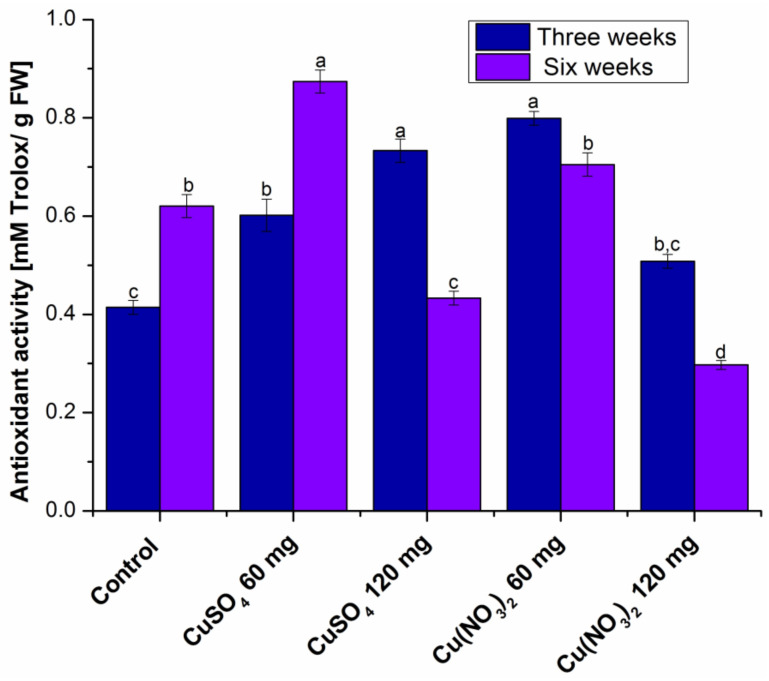
The antioxidant activity (DPPH) of the analyzed extracts. Each data point is the mean ± the standard error of the mean of three independent replicates experiments; different letters mean significant differences between the treatment and the control plants, determined by Tukey’s test (*p* < 0.05).

The antioxidant activity of the analysed plants is presented in Figure 3.

In series I of the copper-treated plants, the antioxidant activity is higher than in the control ones, while in series II of the copper-treated plants, compared to the control plants, the antioxidant activity is higher in those treated with 60 mg of copper salt and lower in those treated with 120 mg of copper salt.

### 2.2. PCA Analysis

**Figure 4:**
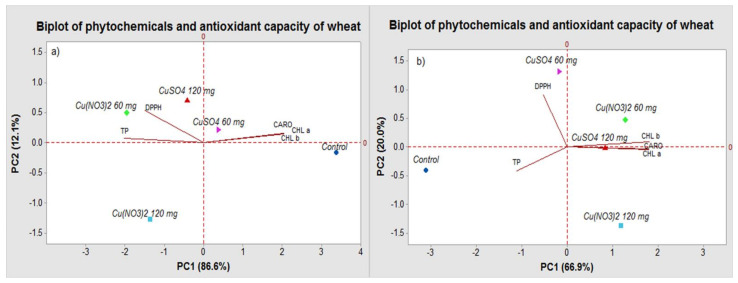
Correlation between the conditions of plant growth, phytochemicals and antioxidant activity of wheat. TP: total polyphenols; CHL a (chlorophyll a); CHL b (chlorophyll b); CARO (total carotenoids); (**a**) series I; (**b**) series II.

In order to determine the interrelations between the parameters (bioactive compounds and the antioxidant activity) of plants treated with different amounts of CuSO_4_, respectively Cu(NO_3_)_2_, the principal components analysis (PCA) was applied. The PCA generated a set of five principal components, and the results are shown in the biplot from Figure 4.

For the series I of plants, the first principal component (PC1) had the highest eigenvalue of 4.33, explaining 86.6% of the total variance (98.7%), and the second principal component (PC2) had an eigenvalue of 0.60, explaining 12.1% of the total variance. The PC1 completely separated the plants treated with CuSO_4_ 60 mg from the plants treated with Cu(NO_3_)_2_ 120 mg. This variation was mainly attributable to the positive loadings of assimilating pigments (CHL a, CHL b and CARO). Along the PC2, a distinct separation was observed in the plant grown with CuSO_4_ 60 mg from the plants grown with CuSO_4_ 120 mg, Cu(NO_3_)_2_ 60 mg and control. This separation was mainly driven by the negative loadings of total polyphenols (TP) and antioxidant activity (DPPH).

In series II, the loading data revealed a separation between the control plants and the treated plants, indicating a distinct pattern of seedling growth in response to the different type and concentration of copper salt.

For the series II of plants, the first principal component (PC1) had the highest eigenvalue of 3.35, explaining 66.9% of the total variance (86.9%), and the second principal component (PC2) had an eigenvalue of 1.00, explaining 20.0% of the total variance. The PC1 completely separated the plants treated with Cu(NO_3_)_2_ 60 mg from the control plants. This variation was mainly attributable to the positive loading of chlorophyll b (CHL b). Along the PC2, a distinct separation was observed in the plants treated Cu(NO_3_)_2_ 60 mg from the plants grown with CuSO_4_ 120 mg, Cu(NO_3_)_2_ 120 mg and CuSO_4_ 60 mg. This separation was mainly driven by the positive loadings of chlorophyll a (CHL a) and total carotenoids (CARO).

### 2.3. Transmission Electron Microscopy Analysis of Wheat Leaves

**Figure 5:**
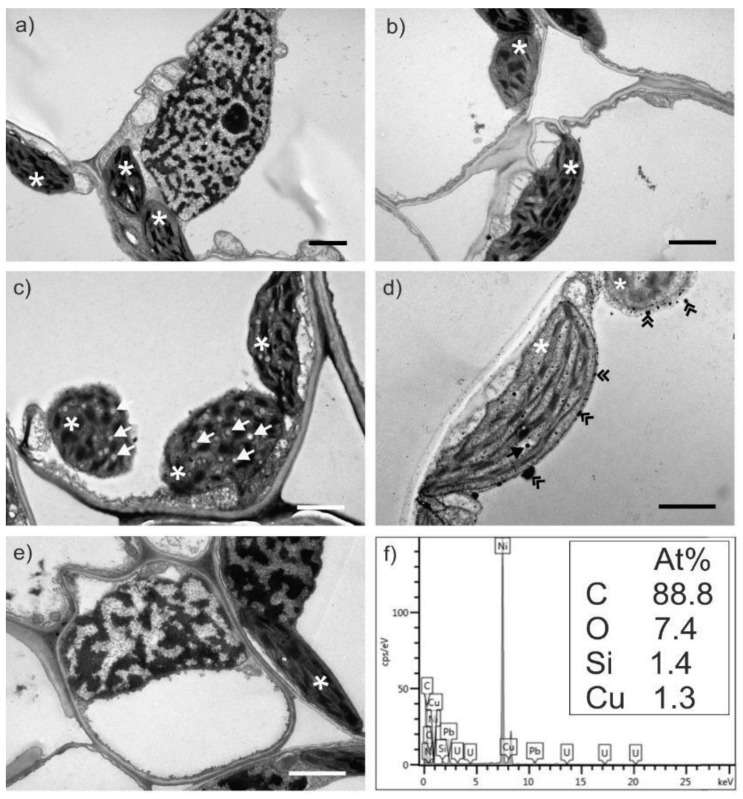
TEM micrographs of *Triticum aestivum* leaves treated with Cu(NO_3_)_2_ solution: 60 mg at three weeks (**a**) and six weeks (**b**), and 120 mg at three weeks (**c**) and six weeks (**d**); untreated controls leaves (**e**); EDX analysis of 120 mg treated plants for six weeks (**f**) indicating Cu accumulations in the electron dense particles (double arrowheads). After six weeks the wheat plants start to develop starch granules (simple arrow) inside the chloroplasts (asterix). All bars are at 2 µm.

**Figure 6:**
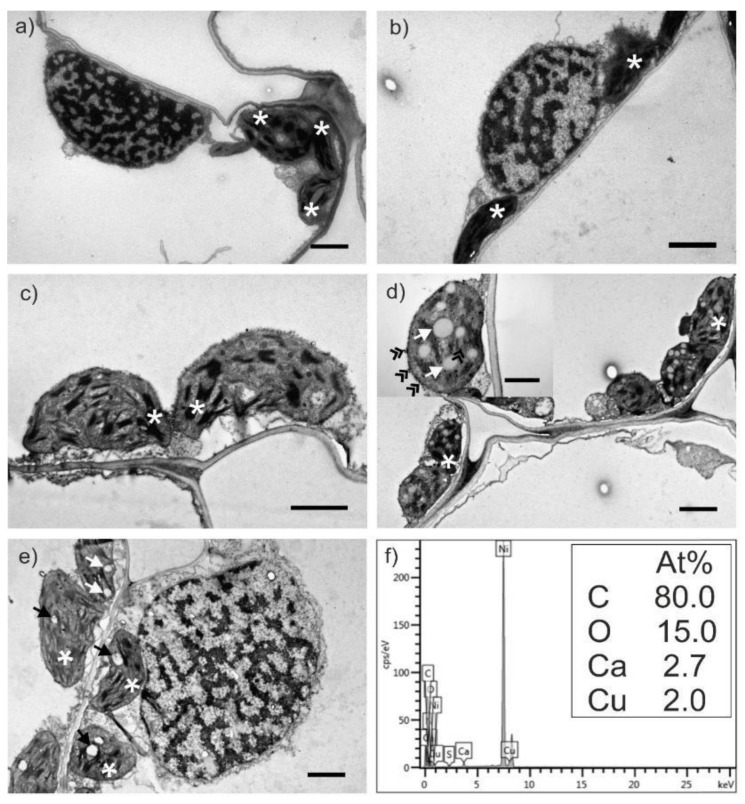
TEM micrographs of *Triticum aestivum* leaves treated with CuSO_4_ solution: 60 mg at three weeks (**a**) and six weeks (**b**), and 120 mg at three weeks (**c**) and six weeks (**d**); untreated control leaves (**e**); EDX analysis of 120 mg treated plants for six weeks (**f**) indicating Cu accumulations in the electron dense particles (double arrowheads). After six weeks the wheat plants start to develop starch granules (simple arrow) inside the chloroplasts (asterix). All bars are at 2 µm; inset with bar at 1 µm (**d**).

The wheat leaves were analyzed through transmission electron microscopy (TEM), to try and determine if the salts affect the ultrastructure of the cells or chloroplasts (Figure 5 and Figure 6). The leaves were harvested at three and six weeks and were compared to an untreated control. At six weeks, the leaves start to develop starch granules [27] and the chloroplasts will slowly transform intro amyloplasts [28]. This phenomenon was observed herein as well. This usually happens during flowering season, which could mean that the salts might favor development.

The plants treated with both solutions at 120 g for six weeks had electron dense accumulations near the tonoplasts (the membrane of the central vacuole). This aspect could indicate the formation of nanoparticles *in planta*, the so-called “biosynthesis”, as the electron dense accumulations were shown to have Cu in their composition detected by elemental dispersive X-ray analysis (EDX, Figure 5f and Figure 6f).

### 2.4. Effect of Cu on Distribution of Other Elements in Wheat

The literature almost entirely lacks articles that study both the effect of nitrate and sulfate of a particular element on plants, much less on wheat. We found only the work of Shtangeeva et al., [29] who studied silver-induced changes in the nutrient and trace element uptake by wheat and amount of the rhizosphere proteins. On the other hand, there are several studies [30,31,32] regarding the effects of Eu, Ce, Ca and Th nitrates on wheat.

Table 1 presents data on the multi-element content of wheat root and the aerial part of the plant. The aerial part was harvested after three and six weeks after sowing and root (farther referred as root) after six weeks. The concentration ranges obtained in our experiment and found in the literature for each of the determined elements were compared. The number of studies covering a wide range of elements is very small, and the research may refer only to the roots or only to the aerial part. For the aerial part the ranges intersect or overlap completely for all elements beside Mo, a reference to which was only found in the literature a single time. The roots present few elements for which the intervals are disjointed. For Na and Br, the data in the literature are higher than those obtained by us, and for V, Cu, Zn, As, Sr and Mo are lower. Given that the data in the literature often cover several orders of magnitude, it is difficult to conclude regarding the influence of the substances applied on the parts of the wheat plant.

The correlation coefficients of the concentration of each chemical element were first calculated to verify the similarity of the influence of sulfate and nitrate on the wheat root and the collected plant after three and six weeks. Then, for each experimental line, the correlation between the root and the green part on the one hand, and between the wheat samples collected after three and six weeks was studied (Table 2).

The element content in roots of wheat grown on soil with CuSO_4_ and Cu(NO_3_)_2_ is mostly positively correlated. Fourteen of 31 elements (Na, Cl, K, Mn, Co, Cu, As, Br, Rb, Ag, Sb, Cs, Ba, Au) have correlation coefficient higher than −0.75. Only a few elements (Mg, Mo, Ta, U) have a negative correlation coefficient but for none of them it is not lower than 0.55.

The two harvesting stages have very different correlations for most of the elements. Only Sm and Th show a high positive correlation between experimental lines. No strong anti-correlation was also found for either harvesting step. The aerial mass reacts differently to external factors at different stages of development.

It can be noticed that the root, at six weeks after sowing, responds similarly to the two external factors applied. Unfortunately, due to the lack of experimental material after three weeks of growth, we cannot conclude that root behavior is the same during the growth period in the presence of sulfate and nitrate.

When wheat has grown in the presence of copper sulfate, the correlation between the root and aerial part is significantly negative (<−0.75) for Na, Cl, Co, As, Br, Cd, Sb and Au, while the strong positive correlation is found only for Cu, Ag and Ba. Wheat collected after three weeks and wheat collected after six weeks has a positive correlation for Co, Br, Mo, Ag, Sb, Ba and Th. For Ca, Sc, Fe, Sr and Cd we notice a strong anti-correlation between the two harvesting stages.

The presence of copper nitrate induces a positive correlation for Na, Sc, Fe, Cs, Ba, and Th, and a negative correlation for Cl, K, Ca, Rb and Ta between the root and aerial part. For the aerial part under the influence of nitrate for three and six weeks we found that for Al, Co, Zn, Rb, Sb, Ba, Th the correlation is positive, and for Na, Mn, Fe, Sr, and Au it is strongly negative.

### 2.5. Cu Content in Wheat

The content of copper in the roots steadily increased with an increase in the amount of the applied substance, regardless of its type. If after three weeks it can be said that both substances had the same effect on wheat roots, after six weeks, the copper content in roots treated with copper nitrate is 55% higher than that of the roots treated with copper sulfate.

**Figure 7:**
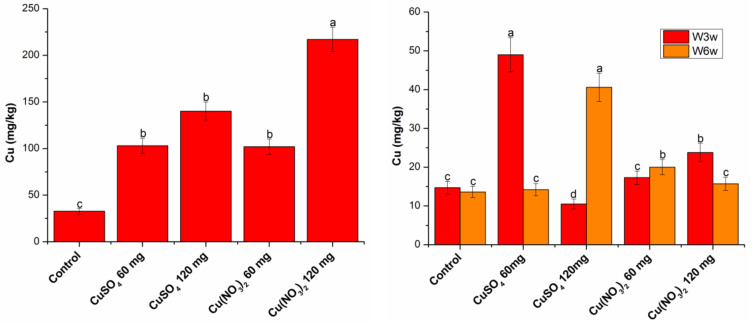
Copper content in root (**left**) and aerial part (**right**) when different concentrations of CuSO_4_ and Cu(NO_3_)_2_ were applied; different letters mean significant differences between the treatment and the control plants, determined by Tukey’s test (*p* < 0.05).

From Figure 7 it can be seen that after three weeks of exposure to copper nitrate and six weeks of exposure to copper sulfate, the copper content in the green part of the plant grows steadily with the increasing concentration of the substance. Three weeks of exposure to copper sulfate 60 mg resulted in a rapid increase in copper levels, while exposure to copper sulfate 120 mg resulted in a decrease in copper levels compared to control. Six weeks of exposure to copper nitrate 60 and 120 mg has a similar character, but the increase is not so sharp, and the copper content at 120 mg of copper nitrate is not lower than the control.

Zuverza-Mena et al. [40] have reported that the uptake of Ca, Fe and Zn competed with the Cu ion in *Coriandrum sativum* under the influence of copper nanoparticles/compounds. However, in the present study, the accumulation of those elements showed no univocal relationship with the Cu ion. Exposure to CuSO_4_ after three weeks led to a high competitiveness of Cu with K, Sc, Mn, As, Rb, Mo, Ag, Cd, Cs and Ba, but after six weeks, only Cs still competed with the Cu ion. Cu(NO_3_)_2_ exposure for three weeks had a negative impact on the accumulation of Na, Sc, Fe, AS, Cd, Cs, Sm, Gd, Ta and Th, while a longer exposure proved that Mg, K, Cr and Rb competed with the Cu ion.

Soil-to-shoot transport is significantly lower compared to control for both copper nitrate concentrations applied, regardless the exposure period. The use of high dose copper sulfate also reduces soil-to-shoot transport after both three and six weeks of exposure, while low dose copper sulfate favors a slight increase in bioaccumulation factor compared to control after six weeks of exposure, but reduces it after three weeks. Transport from root to shoot is also enhanced by using only a small dose of copper sulfate, while high dose copper sulfate and the use of copper nitrate leads to a decrease of TF. The bioaccumulation factor after six weeks is lower than after three weeks regardless of the dose and type of substance applied, with one exception: the use of copper sulfate at low dose for which the factor is higher after six weeks than after three but still lower than three-week control.

### 2.6. Bioaccumulation Factor and Translocation Factor

The capacity of plants to uptake and accumulate metals varies with the plant species and metal type. Brooks et al. [41], first defined hyperaccumulators as plants containing >0.1% of Ni in plant tissues in 1977, and in 1981 Baker [42] suggested to classify plants as accumulators and excluders. The characteristics for classification were gradually improved with the bioaccumulation factor and the translocation factor introduced by McGrath and Zhao and Yoon et al. in 2003 and 2006, respectively [43,44]. The BF and TF higher than one were usually taken to indicate that the plant species could act as a hyperaccumulator [45,46].

The bioaccumulation factor is defined as a measure of the ability of the plant to take up and transport certain chemical elements to shoots, which are parts that are usually harvested. Root-to-shoot transport measured by the translocation factor is one of the key components of elemental hyperaccumulation and, in theory, reveals a reduction in element sequestration in root vacuoles and/or increased xylem loading.

We approached the interpretation of the data from several points of view. First, we compared the values obtained for each of the factors in the presence of the two salts: CuSO_4_ vs. Cu (NO_3_)_2_, CuSO_4_ vs. control and Cu(NO_3_)_2_ vs. control, for each dose applied. The second line of interpretation tries to identify those elements for which the transport of root-shoot and substrate-shoot are simultaneously either stimulated or inhibited by the presence of the two substances.

With rare exceptions, most plants have a bioaccumulation factor for heavy metals and metalloids of less than 1. Even plant species that were initially reported to have a great potential for decontaminating soils polluted with heavy metals finally revealed bioaccumulation factors often less than 0.2 [47].

The range of values of bioaccumulation factor for the studied samples is very wide and covers five orders of magnitude from 0.001 for Al and Sm to 27.9 for Au (Table 2). Even though that for several essential elements, such as Cl, K, Zn, Mo, BF shows values above 1, for some other essential elements, such as Mg, Ca, Mn, Fe, wheat shows a low ability to absorb and transport from the substrate to shoots. Wheat has a bioaccumulation factor for Br and Rb about 1. Gold deserves a special attention because the values of bioaccumulation factor varies from 1.8 and 27.9.

Comparing the influence of the two types of substances on BF resulted in different doses and exposure time, and we can assume that, in the early stage of plant formation, CuSO_4_ allows better absorption and transport to shoots for most elements than Cu(NO_3_)_2_ when a low concentration is applied. At higher doses, for the same period, Cu(NO_3_)_2_ is more permissive. For longer periods, the influence of applied substances is the same but not so obvious.

The comparison of BF exposed wheat with control suggests that both applied substances suppress the uptake and transport of the most elements in the beginning of growth, while a longer exposure seems be better managed by the plants.

In our experiment, the translocation factor was only determined for six weeks of exposure, as root samples were only available after six weeks (Table 2).

As in the case of bioaccumulation factor, the range of values of the translocation factor for the studied samples is quite wide, but covers only four orders of magnitude, from 0.028 for Al to 11.6 for Cl (Table 2). The highest values are shown by some essential elements as Cl, K and Mo, which are joined by Au, Ag, Ba, Br, and Rb.

At small doses, the transport of a large number of elements is more active or the same in the presence of sulphate than nitrate, while at high doses, the number of such elements is limited. The influence of the two types of substances is important not only by direct comparison between the two types of experimental groups but also by comparison with the control samples. Comparing the influence of CuSO_4_ and Cu(NO_3_)_2_ in regard to the control, we ascertain that at low concentrations CuSO_4_ limits the transport from root to shoot of a smaller number of elements than Cu(NO_3_)_2_, while at higher concentrations the translocation process is much more limited in the case of CuSO_4_ as opposed to Cu(NO_3_)_2_, which seems to be more permissive in this regard.

After six weeks of exposure at low doses, wheats ability to transfer Na, Al, K, Cr, Co, Cu, Zn, As, Rb, Sr, Au from both soil to shoot and root to shoot is higher when CuSO_4_ uses rather than Cu(NO_3_)_2_ and lower for Ca, Sc, Mn, Fe, Br, Cs. At higher doses both factors were higher after the application of CuSO_4_ only for K, Sc, Co, Rb, Cd, Cs ad Th, and lower for Mg, Al, Cl, Ca, Mn, Cu, Sr, Mo, Ba, Au. It is obvious that increasing the dose does not only affect K, Co, Ca and Mn, on which the application of CuSO_4_ and Cu(NO_3_)_2_ has the same influence when both 60 mg and 120 mg of the substance were used.

For the most of the elements (Na, Mg, Al, K, Sc, Cr, Fe, Co, Cu, As, Br, Rb, Sr, Sb, Sm), the treatment with 60 mg leads to a more intense transfer both from soil to shoots and from roots to shoots compared to the control, while the same dose of Cu(NO_3_)_2_ has the same effect only for Na, K, Sc, Fe, Zn, Br, Rb, Sb, Cs and Sm. It seems that at small doses, the transfer of Na, K, Sc, Br, Rb, Sb and Sm will be favored to a different extent by the application of both substances. A decrease of both TF and BF compared to control was noted for Ca and Mn when CuSO_4_ (60 mg) is applied and for Cu, Mo, Ag, Ba, Au when Cu(NO_3_)_2_ is used.

For Na, K, Sc, Cr, Fe, Co, Zn, Br, Sb, Cs, Sm the treatment with 120 mg leads to a more intense transfer both from soil to shoots and from roots to shoots compared to the control, while the same dose of Cu(NO_3_)_2_ has the same effect for Na, Ca, Fe, Co, Zn, Br, Sr, Sb, Cs, and Sm. The addition of 120 mg CuSO_4_ reduced both transport processes compared to control in case of Mg, Al, Cl, Ca, Mn, Cu, Mo, Ba, and Au, while the addition of 120 mg Cu(NO_3_)_2_ reduces simultaneously TF and BF for Cu and Au, only. We note that at high doses, CuSO_4_ and Cu(NO_3_)_2_ have similar influence to both BF and TF for Na, Fe, Co, Zn, Br, Sb, Cs, and Sm by enhancing and Cu and Au by suppression.

As was previously mentioned, the literature is very poor with regard to data on the multielement content of wheat. Based on the mean values reported by Shtangeeva et al. [29], for wheat root and leaves in control samples and in wheat grown with AgNO_3_ and Ag_2_SO_4_, we calculated the translocation factor. The translocation factor value for K is almost 2, which is close to our values. In both studies we found that the TF for Ca and Mn is higher when nitrate is applied than when sulfate is used, while sulfate induces a higher TF for Na. Other reported elements (Al, Cr, Mg, Zn) show quite similar values in these two experiments. The differences are quite large in the case of Ag and Cu, but this can probably be explained by the fact that Ag and Cu are the elements of specific interest and added to the substrate in a significant amount.

Using data reported by Shtangeeva et al. [30], Shtangeeva et al. [31] and Shtangeeva, I. [32], the translocation factor was also determined. We have paid attention to those elements among those reported in the literature for which the translocation factor increases or decreases without a doubt in relation to the control regardless of the applied nitrate dose. Thus, we noticed that the application of Th, Ce and Eu nitrate also led to an increase in the translocation factor of K, Fe, Co as well as Cu nitrate, the absolute values of the factor being also comparable with our experimental data. In the case of Au, the application of Th, Ca, Eu and Ca + Eu leads to the decrease of the TF value as in our experiment, the absolute values being subunitary. For none of the elements available in the literature is there a total coincidence of the influence of Th, Ce, Eu, Ca, and Cu nitrate.

### 2.7. Cluster Analysis

A cluster analysis was applied to experimental data to give a better insight into the uptake of elements by plants and to assess the contribution of specific factors that may have an effect on plant behavior.

**Figure 8:**
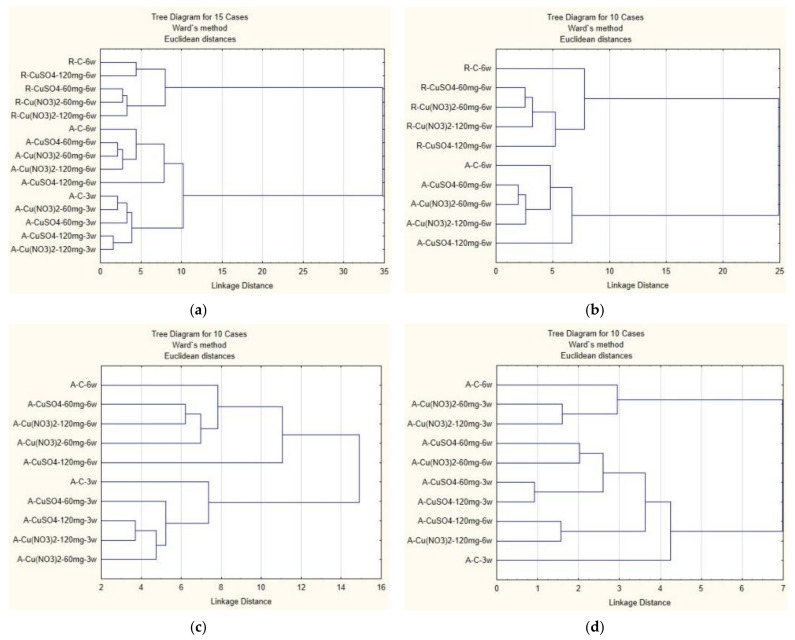

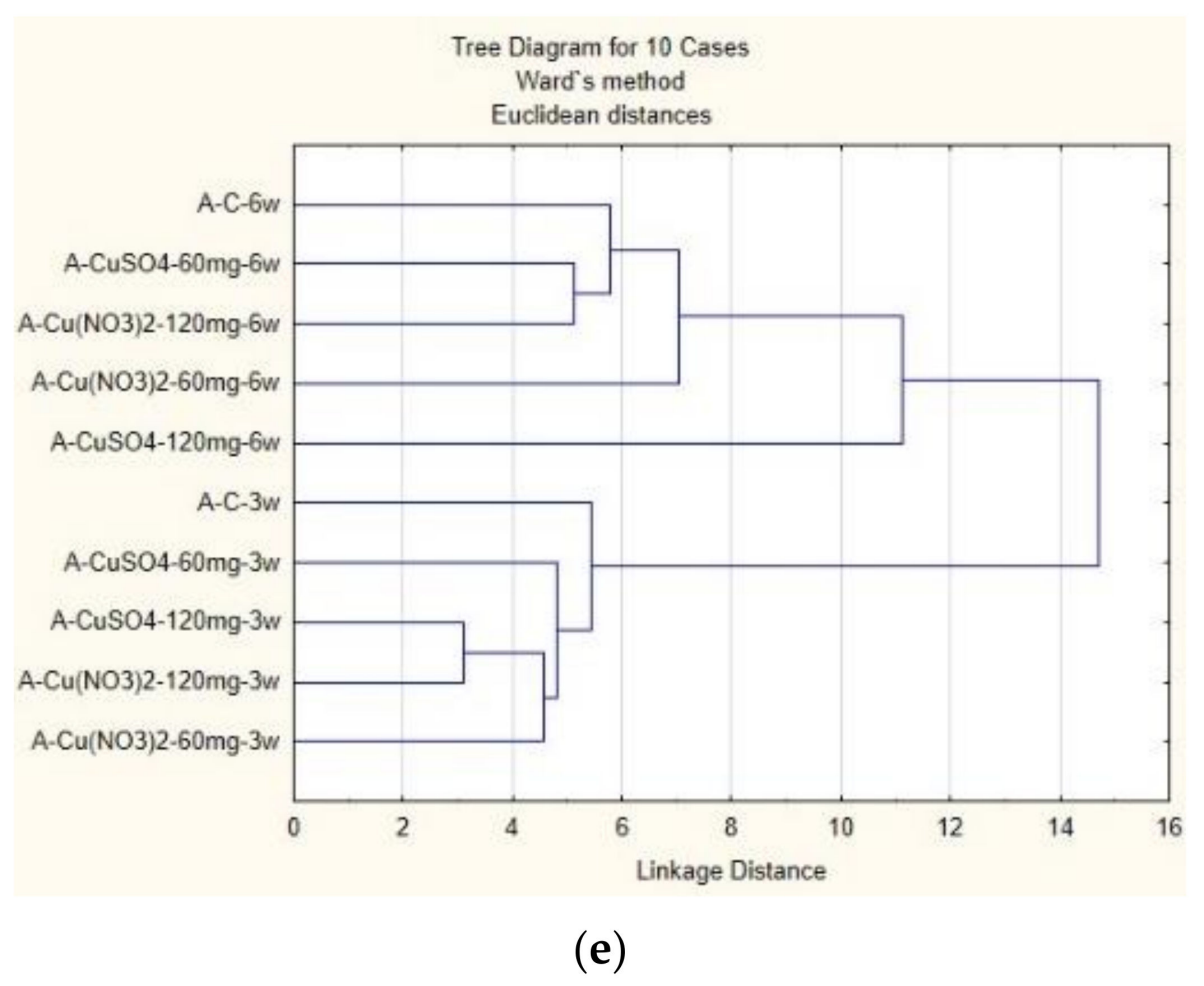
Hierarchical clustering dendrograms for the analysis of the chemical elements that are present both in roots and in three and six weeks aerial part (**a**), the analysis of chemical elements that are present in roots and the aerial part of six weeks only (**b**), the analysis of chemical elements and bioactive compounds aerial part (**c**), for the analysis of biological parameters, aerial part (**d**) and for the analysis of chemical elements, aerial part (**e**).

Figure 8a illustrates the results of cluster analysis based on the content of elements in all samples: the root and the aerial part of the plant. Root and aerial part samples fall into different clusters. The cluster containing the aerial part is also divided into two clusters according to the level of plant development, which coincides with the exposure time. It can be concluded that the differences between the roots and the aerial part of the plants are more significant than other parameters, and the exposure time becomes significant within the aerial part of wheat. The grouping of samples within two clusters of the aerial part is mainly determined by the content of the applied salt.

If we narrow down the analysis to the samples with the longest exposure (root and aerial part), we again see (Figure 8b) that different parts of the plant are grouped into different clusters, and these clusters are almost identical for both parts. The strongest factor on bunching the samples within a cluster seems to be the content of the applied salt and not its type.

Figure 8c–e shows the results of cluster analysis of the aerial part of plants grown in soil with the addition of various copper salts. The results of the cluster analysis presented in Figure 8c were performed based on all available parameters (the abundance of 25 chemical elements and five biocompounds), while the results shown in Figure 6e and Figure 8d are based only on biocompounds and elemental abundance, respectively. The samples are rather well separated into two groups: plants grown for three weeks and plants grown for six weeks. Both clusters contain both plants grown in soil enriched with CuSO_4_ and plants grown in soil enriched with Cu(NO_3_)_2_.

It is easy to see that Figure 6e and Figure 8d are almost identical. The addition of biocompounds changed the “six weeks” cluster with respect to the relationship between the subcluster (S-60 plus N-120) with N-60 and control, respectively. It can be assumed that the difference is due to biocompounds.

As can be seen from Figure 8d, the grouping of samples is not subject to any of the expected factors: the period of exposure, the type or content of the applied compound. While six-week-old samples are grouped by compound content, three-week-old samples are grouped by compound type. The resulting clusters are asymmetric in terms of the number of samples that fall into each of the two clusters.

### 2.8. Correlation between Bioactive Compounds and Elemental Distribution under the Influence of Sulfate and Nitrate

In Table 3 we present correlation coefficients between bioactive compounds and elemental content under the influence of copper sulfate and copper nitrate.

Ca and Mn are negatively correlated with chlorophylls regardless of the substance applied and the time of exposure, but for Mn in the older wheat, the anticorrelation is stronger.

Fe, Sc, Cd, Cs, Sm are correlated with chlorophylls regardless of the substance applied and the time of harvest, but when the wheat is less time exposed, the correlation is stronger for Fe, Sc and Cs and weaker for Cd.

Zn, Br, Ag and Sb mutually relate with chlorophyll at 6w of harvesting and anticorrelate with after 3w of growth regardless of the substance applied.

Mo, Ba and Th present a negative correlation with chlorophyll after 6w of growth and correlate at 3w of harvesting. When CuSO_4_ is applied the correlation with K is positive, while when Cu(NO_3_)_2_ is applied it is negative.

There are several elements that correlate (Al, Sr, Au, Ca) or dissociate (Sc, Fe, Cd, Sm) with the total polyphenol content regardless of the age of the wheat plant and the substance added. On the other hand, the correlation of some elements with the total polyphenol content differs antagonistically depending on the exposure period. Mg, As, Mo and Ag are negatively correlated with TP after 3w of exposure, while after 6w, the correlation changes to positive. A longer exposure leads to a negative correlation for Zn, Br, Sb and Ba, despite the fact that after the first three weeks, the correlation was positive. A difference in correlation related to the substance applied to the substrate is observed for Na only. When CuSO_4_ is applied the correlation with Na is positive, while when Cu(NO_3_)_2_ is applied it is negative.

The variation in antioxidant capacity is closely correlated with the change in Cl content for both experimental lines and is antagonistic to variations in Cd, Cs and Sm content. After three weeks of exposure the correlation is negative for Ba but after six weeks it becomes positive. The type of salt used influences the correlation of several elements. The variation Al, Co, Br and Ba correlates positively with DPPH when CuSO_4_ is used while the use of Cu(NO_3_)_2_ induces a negative correlation. In the case of K, it is exactly the opposite. A positive correlation of K and Br with DPPH was reported also by Soran et al. [39], when the influence of the TiO_2_ nanoparticles on bioactive compounds and the elemental content of wheat was studied by means of addition of the nanoparticles to the growing substrate. The anticorrelation of Al, Mg, Ca and Mo with chlorophylls and carotenoids content and correlation with antioxidant capacity was also reported by Lung et al. [47] when wheat was grown six weeks in a substrate amended with biogenic and chemically obtained CuO NPs.

## 3. Materials and Methods

### 3.1. Chemicals and Materials

CuSO_4_·5H_2_O and Cu(NO_3_)_2_·3H_2_O used for treating plants were purchased from Merck (Germany). The ultrapure water for watering plants was obtained with a Merck Millipore Direct-Q UV3 system (Darmstadt, Germany).

Acetone used for assimilating pigments extraction from wheat plants was obtained from Chimopar, Romania, Folin-Ciocalteu reagent, gallic acid, anhydrous carbonate, 2,2′-Diphenyl-picrylhydrazyl (DPPH) and 6-hydroxy-2,5,7,8-tetramethylchroman-2 carboxylic acid (Trolox) used for total phenolic content quantification and antioxidant capacity determination were sourced from Sigma-Aldrich, Germany, and the ethanol used for obtaining extracts was purchased from Chimopar, Romania.

The seeds of wheat were from the Agricultural Research and Development Station at Turda-Cluj.

The ultrathin sections obtained were contrasted with lead citrate and uranyl acetate and examined with a transmission electron microscope (TEM) Jeol JEM 1010 (JEOL, Tokyo, Japan). The samples were analyzed with TEM Hitachi HD2700 (Hitachi, Tokyo, Japan) cold field emission, operated at 200 kV and coupled with an EDX detector (Oxford Instruments, Oxford, UK, AZtec Software, version 3.3) used for elemental detection. All reagents used for this part of the experiment were acquired from Sigma Aldrich (Merk, Bucharest, Romania).

### 3.2. Plant Growth Conditions

Ten ANDRADA autumn wheat (*Triticum aestivum* L.) seeds were sown at a depth of 1 cm in plastic pots containing 400 g of garden substrate with active humus and fertilizer for six weeks (Agro CS, Slovakia, 50 L). Control wheat was sown only in garden substrate, while the others wheat plants were sown in garden substrate mixed with 60 mg (150 mg/kg) or 120 mg (300 mg/kg) of CuSO_4_·5H_2_O, respectively Cu(NO_3_)_2_. Three pots were placed for each treatment and the plants were grown in a climatronic room (capacity 256 L, Memmert GmbH, Germany) under controlled conditions of light (for 12 h from 24 h), temperature (day/night temperature cycle of 20/10 °C) and humidity (60%). The plant watering was done once every three days with 75 mL of ultrapure water and the plants were harvested and prepared for further analysis at three and six weeks, respectively.

### 3.3. Extraction and Analysis of Assimilating Pigments

0.5 g of freshly ground wheat was mixed with 20 mL of acetone and centrifuged for 10 min at 7000 rpm. 20 mL was added to the remaining plant and stirred on a shaker for 30 min at 300 rpm, at room temperature, after which it was centrifuged for 10 min and the supernatant was decanted. The operation was repeated with another 10 mL of acetone until the plant material discolored. The solutions were combined in the same bottle and were analyzed.

The analysis of chlorophyll a, chlorophyll b and carotenoids from the extracts were done by UV-VIS spectroscopy. In this regard, the absorption spectra of the extracts in the wavelength range 400–750 nm were recorded using a T80 UV-VIS Spectrophotometer (PG Instruments Limited) and the concentrations for chlorophyll a (c_a_), chlorophyll b (c_b_) and total carotenoids (c_(x+c)_) were calculated from the following formulas [48]:

c_a_ (mg mL^−1^) = 11.24 × A_661.6_ − 2.04 × A_644.8_. (1)

c_b_ (mg mL^−1^) = 20.13 × A_644.8_ − 4.19 × A_661.6_. (2)

c_(x+c)_ (mg mL^−1^) = (1000 × A_470_ − 1.90 × c_a_ − 63.14 × c_b_)/214. (3)

### 3.4. Obtaining and Characterizing of Alcoholic Extracts

Over 0.5 g of fresh plant (FW) was added to 7.5 mL 60% ethanol. The mixture was subjected to ultrasonic-assisted extraction using an Elma Transsonic T ultrasonic bath for 30 min at room temperature and then centrifuged at 7000 rpm for 10 min. The supernatant was decanted and stored in the refrigerator at 4 °C until analysis.

#### 3.4.1. Determination of Total Polyphenol Content

The content of total polyphenols was determined by the Folin-Ciocalteu method according to the protocol presented by Ivanova et al. [49].

For this purpose, 1 mL of extract was added to a 10 mL volumetric flask containing 5 mL of double distilled water. To this mixture was added 0.5 mL of Folin-Ciocalteu reagent, the contents were mixed and after 3 min of standing, 1.5 mL of Na_2_CO_3_ (5 g L^−1^) was added. The volume of the flask was adjusted with double-distilled water. After keeping the samples at 50 °C (in a water bath) for 16 min in closed flasks, followed by cooling to room temperature, the absorbances were read in relation to the blank sample (double-distilled water) at a wavelength of 765 nm.

The calibration curve was constructed using standard gallic acid solutions in the range 0.001–0.800 mg mL^−1^, obtained by successive dilutions with double-distilled water starting from a solution of concentration 1 mg mL^−1^.

#### 3.4.2. Determination of the Antioxidant Capacity

The antioxidant capacity was evaluated after a slightly modified procedure, reported by Brand-Williams and collaborators [50]. Thus, 0.01 mL of extract was added to 3.9 mL of DPPH-2,2 diphenyl-picryl-hydrazyl radical solution (0.0025 g/100 mL methanol) and after 10 min in the dark the absorbance of the mixture was measured at 515 nm compared to the control sample (0.01 mL extract added to 3.9 mL methanol). The results were calculated from the calibration curve and expressed in mM Trolox/1 g plant.

The calibration curve was plotted for different concentrations of Trolox (0–400 µM) at a wavelength of 515 nm.

### 3.5. Transmission Electron Microscopy Analysis of the Wheat Leaves

The samples were collected after treatment and prepared according to previous studies [47]. Briefly, the leaves were placed in glutaraldehyde fixator (2.7%) for 1.5–2 h, after which they were washed in phosphate buffered saline (PBS 0.1 M) four times. The second fixator (osmium tetroxide) was left on the samples for 1 h and washed afterwards three times with PBS. As embedding resin Epon 812 was used, prior dehydration with acetone in increasing concentrations (30–100%).

### 3.6. Elemental Content of the Wheat Biomass and Soil Substrate

The elemental content of the wheat samples and soil substrate was determined by neutron activation analysis (NAA) at the pulsed fast reactor IBR-2 (FLNP JINR, Dubna, Russia). The information about irradiation of wheat and soil samples can be found in Lung et al. [47]. The reference materials used for analysis included the following: Trace Elements in Soil (2709), Pine needles (1575a), Calcareous soil (690CC), Marine sediment (433) and Trace Elements in Coal (1632c) were irradiated in the same conditions with samples. The data processing was performed using the software developed at FLNP JINR [51] and the results are expressed on a dry weight basis.

### 3.7. Statistical Analysis

The results from the study are presented as the mean three replicate samples for each concentration ± SD. One-way analysis of variance (ANOVA) followed by Tukey’s test performed with Minitab 17 (Minitab Ltd., Coventry, UK) was used to evaluate the statistically significant differences between groups (*p* < 0.05), were tested for significance using one-way ANOVA. Statistical significance was considered at 95% confidence intervals.

Moreover, PCA and hierarchical cluster analysis (HCA) was performed using Minitab 17 (Minitab Ltd., Coventry, UK).

The bioaccumulation factor (BF) and translocation factor (TF) were calculated as follows [43]: BF = C_shoot_/C_soil_ where C_shoot_ is the heavy metals concentration in shoots. And C_soil_ is the heavy metals concentration in soils [44]. TF = C_shoo_t/C_root_.

## 4. Conclusions

The main objective of this study was to determine the effect of two copper salts, namely CuSO_4_ and Cu (NO_3_)_2_, on bioactive compounds and elemental content from wheat, as well as on the ultrastructure of wheat leaves.

Thus, there was a decrease in series I plants and an increase in series II plants in terms of the amount of chlorophyll a, chlorophyll b and total carotenoids in plants grown in the presence of copper salts compared to control plants. Regarding the amount of total polyphenolic compounds, in series I of plants it increases, while in series II of plants it decreases in those treated with copper salt compared to the control plant.

Regarding antioxidant activity, in the first series of plants it increases compared to the control, and in the second series of plants the antioxidant activity is higher in those treated with 60 mg of copper salt and lower in those treated with 120 mg of copper salt compared to the control plant.

At low doses, the ability to transfer Na, K, Sc, Br, Rb, Sb and Sm from soil to shoot and from root to shoot will be favored to varying degrees by the use of both substances.

The impact of both salts at high doses had the same effect on the bioaccumulation factor and the translocation factor for Na, Fe, Co, Zn, Br, Sb, Cs, and Sm—enhancement and for Cu and Au—suppression.

No significant damages were induced in the leaves of the wheat plants treated with the selected salts. However, at six weeks it seems that the plant starts to transform the salts into nanoparticles, as indicated by EDX analysis.

## Figures and Tables

**Table 1:** Element content (minimum and maximum values) in soil and wheat after three and six weeks of exposure (mg/kg).

	Exerimental Data for Wheat					Literature Data for Wheat		References
	Soil 3w	Soil 6w	Root	A 3w	A 6w	Root Control	A Control	
Na	3020–7700	2820–4600	1040–1650	380–410	390–470	2900–7710	250–10,000	[29,33,34]
Mg *	5.1–13	9.6–18.7	2.6–3.3	2.0–2.9	0.49–2.6	0.07–2.7	0.079–2.2	[29,33,35,36]
Al *	7.9–24.4	15.4–23	0.26–0.71	0.070–0.084	0.020–0.071	0.118–0.314	0.0059–0.067	[29,36,37]
Cl *	0.59–1.4	0.72–1.5	1.3–4.3	9.3–12.6	2.5–23	3.4–5.4	5.5–27	[33,35,37]
K *	5.9–9.2	6–7.5	49–60	101–108	126–148	3–351	8.6–657	[32,33]
Ca *	33–50	46–61	4.7–6.7	3.2–4.9	0.74–6.5	0.26–5.6	0.05–5.1	[29,33,35,36]
Sc	2.6–4.4	2.5–4.2	0.058–0.19	0.014–0.026	0.011–0.039	0.015–0.18	0.01–0.15	[34,37]
V	14–32.9	23.1–38.9	0.63–1.5	ND	ND	0.071	0.04	[37]
Cr	14.3–31	14.2–24.4	1.5–8.2	ND	1.2–4.9	0.68–12.3	0.41–6.6	[29,37]
Mn	350–630	520–580	25.4–48	24.2–35	3.2–21.4	6.9–63	2–44	[29,33]
Fe *	8–13.8	7–12.9	0.3–0.68	0.12–0.17	0.12–0.17	0.030–0.84	0.15–1.42	[34,35,36,37]
Co	3.5–5	3.1–5	0.35–0.56	0.039–0.054	0.048–0.096	0.002–0.88	0.013–0.08	[32,34]
Cu	37.6–182	60.4–262	32.8–217	13.6–40.6	10.5–49	0.08–12	0.14–104	[32,33,37]
Zn	66–101	69–80	431–538	80–92	50–67	25.9–329	35.7–200	[33,34,35]
As	4.5–5.8	3.9–5.9	3.6–5	0.67–0.84	0.66–0.87	0.042–0.58	0.01–0.43	[37,38]
Br	8.1–11	10.3–14.1	5.42–8.5	9.9–11.1	4.88–6.8	12.4–33	7.5–11	[34,38]
Rb	26–42	21–34	27–34	43–46	44–48	5.2–61	6.8–88	[34,38]
Sr	96–154	90–123	18.8–23.7	11.9–13.4	9.6–18	0.24	0.1–37.4	[39]
Mo	1–1.4	0.81–3.8	0.58–0.88	1.7–2	1.6–2.5	0.31	0.47	[37]
Sb	2–2.8	1.8–3.2	0.19–0.3	0.037–0.076	0.018–0.048	0.02–1.94	0.02–0.98	[32,37]
Cs	1.2–2.1	1.1–1.5	0.027–0.12	0.0076–0.019	0.017–0.05	0.014–0.77	0.01–0.26	[37,38]
Ba	240–410	230–330	24–39	25–33	28–53	12.9–33	9.8–158	[36,37]
La	8.7–14.4	7.2–11.9	0.18–0.42	ND	ND	0.3–0.36	0.07	[34,38]
Sm	1.6–2.5	1.3–2.1	0.028–0.074	0.0036–0.019	0.0021–0.016	0.04–0.1	0.008–0.01	[34,38]
Ta	0.27–0.44	0.22–0.38	0.0087–0.98	0.013–0.021	ND	<0.2	<0.2	[38]
Au	0.0016–0.032	0.0019–0.0068	0.005–0.044	0.013–0.057	0.044–0.066	0.008–0.02	0.005–0.01	[34,39]
Th	2.5–4	2.2–3.4	0.066–0.14	0.010–0.017	0.0146–0.16	<0.02–0.09	<0.007–<0.02	[34,38,39]
U	0.81–1.32	0.74–1.14	0.17–0.28	ND	ND	na	<0.03	[38]

* content in g/kg; ND—not detected; na—not available; A—means aerial part of wheat plant.

**Table 2:** Correlation of elemental content in various parts of wheat grown in soil with the addition of CuSO_4_ and Cu(NO_3_)_2_.

	CuSO_4_ vs. Cu(NO_3_)_2_			CuSO_4_		Cu(NO_3_)_2_	
	R−R	A 3w−3w	A 6w−6w	R−A 6w	A 3w−6w	R−A 6w	A 3w−6w
Na	**0.96**	**0.96**	0.00	−0.78	−0.28	0.81	**−1.00**
Mg	−0.52	**1.00**	0.27	0.32	−0.53	−0.47	0.74
Al	0.56	**−0.99**	0.56	−0.25	−0.31	−0.25	**0.92**
Cl	**0.97**	0.57	**0.99**	**−0.95**	−0.69	−0.75	0.03
K	**0.97**	−1.00	**0.92**	−0.54	−0.56	**−0.94**	0.13
Ca	0.60	−0.19	0.85	−0.33	**−0.99**	**−0.97**	−0.45
Sc	0.74	−0.54	0.78	0.03	−0.81	**0.99**	−0.66
V	0.69	ND	ND	ND	ND	ND	ND
Cr	0.63	−0.01	ND	ND	ND	ND	ND
Mn	**0.96**	0.49	**−0.96**	0.58	−0.46	−0.57	−0.76
Fe	0.60	−0.11	**0.97**	0.39	−0.81	**1.00**	−0.70
Co	**1.00**	−0.73	−0.16	**−0.99**	**1.00**	0.36	0.76
Cu	**0.95**	−0.34	−0.18	0.78	−0.57	0.18	0.09
Zn	0.20	**0.92**	0.72	−0.29	0.73	−0.65	**0.92**
As	0.80	**−1.00**	0.65	**−0.90**	0.39	−0.48	0.51
Br	**0.94**	**0.98**	0.16	−0.87	**0.93**	−0.33	0.66
Rb	0.87	**−0.94**	**0.94**	−0.19	−0.76	−0.87	0.76
Sr	0.48	0.23	−0.17	−0.14	**−0.99**	−0.19	**−0.97**
Mo	−0.58	−0.61	0.87	0.24	**0.99**	0.19	−0.24
Ag	**1.00**	0.19	0.75	**0.94**	**0.99**	0.53	−0.36
Cd	0.73	0.83	−0.55	−0.86	−0.85	−0.65	−0.54
Sb	**1.00**	0.77	0.14	**−0.99**	**0.92**	−0.30	**0.94**
Cs	**0.98**	**0.98**	0.56	0.51	−0.67	**0.99**	0.06
Ba	**0.97**	0.71	0.80	**0.98**	**1.00**	**0.98**	**1.00**
La	0.66	ND	ND	ND	ND	ND	ND
Sm	0.30	1.00	0.75	−0.05	−0.72	**0.93**	−0.10
Gd		ND	0.85	ND	ND	ND	ND
Ta	−0.36	ND	0.56	−0.33	ND	**−0.96**	ND
Au	0.81	−0.70	**1.00**	−0.87	0.17	−0.44	−0.84
Th	0.66	**0.99**	0.81	0.35	**0.94**	**0.97**	**0.92**
U	−0.54	ND	ND	ND	ND	ND	ND

*R*^2^ > 0.9 and *R*^2^ < −0.9—bold; *R*^2^ > 0.75—red; *R*^2^ < −0.75—blue. ND—not detected.

**Table 3:** Correlation of elemental content with bioactive compounds in aerial part of wheat grown in soil with addition of CuSO_4_ and Cu(NO_3_)_2_.

	A 6w CuSO_4_					A 6w Cu(NO_3_)_2_					A 3w CuSO_4_					A 3w Cu(NO_3_)_2_				
	Chl_a_	Chl_b_	CARO	TP	DPPH	Chl_a_	Chl_b_	CARO	TP	DPPH	Chl_a_	Chl_b_	CARO	TP	DPPH	Chl_a_	Chl_b_	CARO	TP	DPPH
Ch_a_	1					1					1					1				
Ch_b_	**1.00**	**1**				**1.00**	**1**				**1.00**	**1**				**1.00**	**1**			
CARO	**1.00**	**1.00**	**1**			**1.00**	**1.00**	**1**			**1.00**	**1.00**	**1**			**1.00**	**1.00**	**1**		
TP	**−0.96**	**−0.95**	**−0.97**	**1**		**−0.93**	**−0.96**	**−0.94**	**1**		**−0.99**	**−0.98**	**−0.98**	**1**		**−0.99**	**−0.99**	**−0.99**	**1**	
DPPH	−0.03	0.00	−0.05	0.31	**1**	−0.31	−0.23	−0.28	−0.06	**1**	**−0.98**	**−0.98**	**−0.98**	**0.92**	**1**	−0.67	−0.65	−0.66	0.76	**1**
Na	0.12	0.16	0.11	0.16	**0.99**	0.51	0.58	0.53	**−0.79**	0.66	**−0.95**	**−0.95**	**−0.96**	0.88	0.99	0.47	0.45	0.47	−0.58	**−0.97**
Mg	−0.63	−0.60	−0.64	0.82	0.80	−0.60	−0.53	−0.58	0.26	**0.95**	0.25	0.25	0.21	−0.41	−0.03	**0.99**	**0.99**	**0.99**	**−0.96**	−0.55
Al	−0.28	−0.25	−0.30	0.54	**0.97**	0.02	−0.06	−0.01	0.35	**−0.96**	**−1.00**	**−1.00**	**−1.00**	**0.99**	**0.96**	−0.46	−0.49	−0.47	0.34	−0.35
Cl	−0.72	−0.70	−0.74	0.89	0.71	0.28	0.35	0.30	−0.61	0.83	**−0.99**	**−0.99**	**−0.99**	**1.00**	**0.94**	**−0.98**	**−0.98**	**−0.98**	**0.95**	0.50
K	0.89	0.87	**0.90**	**−0.98**	−0.49	−0.8	−0.82	−0.85	0.61	0.75	0.20	0.20	0.24	−0.03	−0.41	−0.35	−0.33	−0.34	0.47	**0.93**
Ca	−0.79	−0.77	−0.80	**0.93**	0.64	−0.56	−0.62	−0.58	0.83	−0.62	**−0.90**	**−0.90**	**−0.91**	**0.81**	**0.97**	**−1.00**	**−1.00**	**−1.00**	**0.98**	0.61
Sc	0.58	0.56	0.60	−0.79	−0.83	0.49	0.55	0.51	−0.78	0.68	**0.97**	**0.97**	**0.98**	**−0.92**	**−1.00**	**0.97**	**0.96**	**0.96**	**−0.99**	−0.84
Cr	0.69	0.71	0.67	−0.46	0.70	−0.64	−0.57	−0.62	0.31	**0.93**	ND	ND	ND	ND	ND	ND	ND	ND	ND	ND
Mn	−0.88	−0.86	−0.89	**0.98**	0.51	**−0.91**	**−0.94**	**−0.92**	**1.00**	−0.11	−0.06	−0.06	−0.09	−0.11	0.28	−0.38	−0.35	−0.37	0.49	**0.94**
Fe	0.82	0.80	0.83	**−0.95**	−0.59	0.59	0.65	0.61	−0.85	0.59	**0.99**	**0.99**	**0.99**	**−1.00**	**−0.95**	**0.98**	**0.98**	**0.98**	**−1.00**	−0.79
Co	**0.94**	**0.95**	**0.93**	−0.80	0.32	−0.56	−0.62	−0.58	0.82	−0.62	−0.88	−0.88	−0.86	**0.95**	0.75	−0.16	−0.19	−0.17	0.03	−0.63
Cu	0.30	0.33	0.28	−0.02	**0.94**	0.71	0.65	0.69	−0.40	−0.89	−0.68	−0.68	−0.70	0.54	0.82	−0.73	−0.71	−0.73	**0.81**	**1.00**
Zn	0.81	0.83	0.80	−0.61	0.56	**0.99**	**1.00**	**1.00**	**−0.96**	−0.21	**−1.00**	**−1.00**	**−1.00**	**0.98**	**0.98**	−0.85	−0.84	−0.85	**0.91**	**0.96**
As	0.04	0.07	0.02	0.24	**1.00**	−0.24	−0.31	−0.26	0.58	−0.85	**0.94**	**0.94**	**0.95**	−0.86	**−0.99**	**0.94**	**0.93**	**0.94**	**−0.98**	−0.87
Br	**0.96**	**0.97**	**0.96**	**−0.85**	0.24	**1.00**	**0.99**	**1.00**	**−0.91**	−0.36	**−0.75**	**−0.75**	−0.72	0.85	0.59	−0.65	−0.67	−0.66	0.55	−0.13
Rb	**0.99**	**1.00**	**0.99**	**−0.92**	0.09	**−0.94**	**−0.91**	**−0.93**	0.75	0.61	0.87	0.87	0.89	−0.77	**−0.96**	0.53	0.55	0.53	−0.41	0.28
Sr	**−0.99**	**−1.00**	**−0.99**	**0.92**	−0.09	−0.24	−0.32	−0.27	0.58	−0.85	**−1.00**	**−1.00**	**−1.00**	**0.99**	**0.97**	0.03	0.06	0.04	0.10	0.73
Mo	−0.69	−0.66	−0.70	0.86	0.75	−0.29	−0.36	−0.32	0.62	−0.82	0.66	0.66	0.69	−0.53	−0.81	0.88	0.89	0.89	−0.81	−0.23
Ag	**−0.92**	**−0.91**	**−0.93**	**0.99**	0.42	−0.67	−0.72	−0.68	0.89	−0.51	**0.99**	**0.99**	**0.99**	**−0.94**	**−1.00**	0.49	0.52	0.50	−0.38	0.32
Cd	0.76	0.74	0.78	**−0.91**	−0.67	**0.97**	**0.95**	**0.97**	−0.82	−0.52	0.38	0.38	0.41	−0.22	−0.57	0.69	0.67	0.69	−0.78	**−1.00**
Sb	**0.91**	**0.92**	**0.90**	**−0.75**	0.39	**0.93**	0.89	**0.92**	−0.72	−0.64	−0.61	−0.61	−0.58	0.74	0.42	−0.78	−0.79	−0.78	0.69	0.05
Cs	0.34	0.31	0.35	−0.58	**−0.95**	0.38	0.31	0.36	−0.02	−1.00	**0.95**	**0.95**	**0.96**	−0.89	**−1.00**	0.88	0.86	0.87	**−0.93**	**−0.94**
Ba	−0.82	−0.80	−0.83	**0.95**	0.60	**−1.00**	**−1.00**	**−1.00**	**0.94**	0.27	**0.91**	**0.91**	**0.92**	−0.82	**−0.98**	**1.00**	**1.00**	**1.00**	**−1.00**	−0.69
Sm	0.78	0.76	0.79	**−0.92**	−0.65	0.71	0.65	0.69	−0.40	−0.89	**0.98**	**0.98**	**0.98**	**−1.00**	**−0.92**	0.74	0.73	0.74	−0.82	**−0.99**
Gd	ND	ND	ND	ND	ND	ND	ND	ND	ND	ND	**0.99**	**0.99**	**0.99**	**−1.00**	**−0.94**	0.87	0.86	0.87	**−0.93**	**−0.94**
Ta	ND	ND	ND	ND	ND	ND	ND	ND	ND	ND	0.85	0.85	0.84	**−0.93**	−0.72	0.22	0.19	0.21	−0.35	**−0.87**
Au	**−0.92**	**−0.91**	**−0.93**	**0.99**	0.42	0.24	0.16	0.21	0.13	**−1.00**	−0.15	−0.15	−0.11	0.32	−0.07	−0.29	−0.26	−0.28	0.41	0.91
Th	**−0.95**	**−0.96**	**−0.95**	0.83	−0.28	**−1.00**	**−1.00**	**−1.00**	**0.94**	0.28	**1.00**	**1.00**	**1.00**	**−0.99**	**−0.97**	0.89	0.87	0.88	**−0.94**	**−0.94**

*R*^2^ > 0.9 and *R*^2^ < −0.9—bold; *R*^2^ > 0.75—red; *R*^2^ < −0.75—blue. ND—not determined.

## Data Availability

Not applicable.
